# Evaluation of phytochemical and pharmacological properties of seeds of *Momordica charantia*

**Published:** 2020

**Authors:** Sumyya Zahan, Tajbiha-E- Mowla, S.M. Naim Uddin, Mohammed Kamrul Hossain, Afifa Binthe Mannan, Minhajur Rahman, Umay Chen, Tanoy Mazumder, A.H.M. Mazbah Uddin, Sayema Arefin, Md. Saddam Hussain

**Affiliations:** 1 *Department of Pharmacy, University of Chittagong, Chittagong-4331, Bangladesh*; 2 *Department of Pharmacy, Southern University, Chittagong, Bangladesh*; 3 *Department of Genetic Engineering and Biotechnology, University of Chittagong, Chittagong-4331, Bangladesh*; 4 *Department of Botany, University of Chittagong, Chittagong-4331, Bangladesh*; 5 *Department of Pharmacy, Noakhali Science and Technology University, Sonapur, Noakhali-3814, Bangladesh*; 6 *Department of Pharmacy, Mawlana Bhashani Science and Technology University, Santosh-1902, Tangail*

**Keywords:** Momordica charantia, Seeds, In vitro, In vivo

## Abstract

**Objective:**

The purpose of the current study was to investigate the *in vivo* (analgesic, antidiarrheal, neurological, and cytotoxic) and *in vitro* (antioxidant, antimicrobial, thrombolytic and anthelmintic) activity of different fractions of methanolic extract of *Momordica charantia*.

**Materials and Methods:**

The antioxidant property was evaluated by DPPH radical scavenging assay, while antimicrobial activity was examined against three Gram (+) and one Gram (-) bacteria. Thrombolytic and anthelmintic activities were evaluated by using human blood serum and by recording paralysis and death time in earthworm, respectively. Cytotoxic activity was investigated in brine shrimp nauplii. Analgesic and antidiarrheal activities were evaluated in Swiss albino mice and neurological effect was evaluated by open field and Elevated plus-maze test (EPM).

**Results:**

All fractions (n-hexane, carbon tetrachloride and chloroform) possess significant (p<0.05) cytotoxic activity. In case of thrombolytic activity, the highest concentration of methanolic extract produced a remarkable percentage of clot lysis (46.12%). The concentration of 1000 μg/ml produced a significant antibacterial activity against Gram positive *Staphylococcus aureus* and Gram negative *E. coli*. Aqueous fraction at a dose of 400 mg/kg body weight, was found to show promising analgesic activity. In case of antidiarrheal and anthelmintic activity, plant extract showed dose-dependent activity. Methanolic extract and its fractions failed to produce any neurological effect in both methods.

**Conclusion:**

The overall results of the study tend to suggest that the methanolic extract and its fractions have promising pharmacological activities.

## Introduction

From the ancient times, people have used medicinal plants as a potential source of life for the recuperation from major and minor ailments (Hussain et al., 2018). *Momordica charantia* (MC) also commonly known as bitter melon, bitter apple, bitter gourd or karela, belongs to the family Cucurbitaceae (Yoshime et al., 2016). Contemporary scientific research has proven that bitter melon auspiciously possesses different pharmacological activities including antidiabetic, anticancer, antimicrobial, anti-hepatotoxic, antioxidant, antiviral, antiulcerogenic, and larvicidal activities (Anjum et al., 2012).

Several bioactive compounds like alkaloids, tannins, carbohydrates, terpenoids, steroids, flavonoids, etc. naturally exist in medicinal plants and they are responsible to produce definite pharmacological actions in the human body (Prakash et al., 2015; Osman et al., 2014; Lombardi et al., 2017). 

Different nutrient and non-nutrient constituents of the plant materials possess anticancer properties that were established in various *in vitro* and *in vivo* models and by utilizing these constituents and cancer prevention strategies had emphasized (Bulbul et al., 2013). 

Again thrombus or blood clot ends up with severe consequences of thrombolytic issues like acute myocardial and cerebral infraction which can also lead to demise (Khan et al., 2013). Most of the thrombolytic agents possess significant shortcomings including bleeding tendency, limited fibrin specificity and requirement of a large dose to reach the maximum therapeutic effect (Khan et al., 2013). Studies carried out on herbs showed thrombolytic activity and a few remarkable observations have been reported (Basta et al., 2004; Khan et al., 2013; Bulbul et al., 2013). 

Antibiotics are one of the most important weapons to fight against different types of bacteria. Over the past few decades, antibiotics turned out to be less effective because of the emergence of drug resistant bacteria. This has become an essential area of studying newer drugs which are lesser resistant to the bacterial infection (Farnsworth, 1993). 

Pain is associated with potential tissue damage which is responsible to produce unpleasant sensory and emotional experience (John and Rolf-Detlef, 2008). Analgesic compounds act on the CNS and alleviate pain selectively by reducing local inflammatory responses without significant alteration of consciousness (Dewan et al., 2013). 

In Bangladesh, diarrhea is liable to the death of one third of the children (Galheigo et al., 2016; Patzi-Vargas et al., 2015). It has been reported that medicinal plants are efficiently used for the treatment of diarrhea (Dooley et al., 2015). Various antidiarrheal compounds increase its toxicity problems due to the mismanagement or development of resistance (Bulbul et al., 2013). Researchers are highly interested in development of synthetic anthelmintic agents from medicinal plants to minimize all the problems caused by antihelminthic drugs (Bulbul et al., 2013; Bulbul et al., 2013). 

The present study was designed to investigate the phytochemical and pharmacological properties of different fractions of methanolic extract of MC seeds by *in vitro* and *in vivo* bioassays. 

## Materials and Methods

**Selection, collection and preparation of seeds extract**

In the current study, seeds of MC were collected from the local market of Chittagong city, Bangladesh. An expert of Bangladesh National Herbarium located in Mirpur, Dhaka, Bangladesh, ascertained the seeds with accession number- DACB: 93486. After drying, seeds were ground into a rough powder using an appropriate grinder. Then, 550 g of grinded-powdered material was taken in exceedingly clean, flat-bottomed glass instrumentation and soaked in 2500 ml of 98% pure methanol. The container was sealed perfectly with its content and kept for 15 days with occasional shaking and stirring. The whole mixture then underwent through a two-step filtration process; firstly with the help of fresh cotton materials and then by a Whatman filter paper (460 mm x 570 mm) (Bibby RE200, Sterilin Ltd., UK) (Hira et al., 2013).

**Fractionation**

With the help of protocol designed by Kupchan and Tsou and modified method of Van-Wagenen et al., solvent-solvent partitioning was accomplished (Van-Wagenen et al., 1993). Here, 5 g of the crude extract was triturated by 98% methanol. The prepared solution was then fractionated successfully using solvents of increasing polarity, such as n-hexane, carbon tetrachloride and chloroform.

**Collection of earthworms**

Earthworms (*Phertima posthuma*) were collected from moist soil in the campus of Chittagong University (3–5 cm in length and 0.1–0.2 cm in width weighing 0.8–3.04 g). They were thoroughly washed with saline water.

**Experimental animal**

Male and female Swiss-albino mice (*Mus musculus*), age range of 4-5 weeks, were used for the experiment; mice were collected from the Animal Lab of Department of Pharmacy, Jahangirnagar University. Animal experimentations were done according to the guidelines of the Institutional Animal Ethics Committee (ARRP Guideline 22: Guidelines for the Housing of Mice in Scientific Institutions). 

**Experimental design**

During every experiment, forty experimental animals were randomly divided into eight groups of 5 mice. 

During neurological activity test each group received a particular treatment:

Group I: Control (1% v/v Tween-80 in water, 0.5 ml/mice).

Group II: Positive control (diazepam 1 mg/kg body weight)

Group III: Test sample 1 (methanolic extract at the dose of 400 mg/kg body weight)

Group IV: Test sample 3 (chloroform fraction at the dose of 400 mg/kg body weight)

Group V: Test sample 4 (carbon tetra-chloride fraction at the dose of 400 mg/kg body weight)

Group VI: Test sample 5 (n-hexane fraction at the dose of 400 mg/kg body weight) (Chowdhury et al., 2015; Abedin et al., 2018; Sen et al., 2018; Sarkar et al., 2016).

During analgesic activity test: Forty experimental animals were randomly clustered into eight groups as follows: group I, group II, group III (A-B), group IV, group V, group VI, and group VII consisting of five mice in every group. Every cluster received a selected treatment. Before any treatment commenced, every experimental animal was weighed properly and the doses of the test samples and control materials were adjusted accordingly. Mice of each group was marked as M-1=Mice 1, M-2=Mice 2, M-3=Mice 3, M-4=Mice 4 and M-5=Mice 5 (Sarkar et al., 2016).

During antidiarrheal activity: Forty experimental animals were randomly selected and grouped similarly as discussed above the previous paragraph (Abedin et al., 2018).

**Phytochemical evaluation**

The seeds of MC were subjected to preliminary quantitative phytochemical studies for detection of phytochemicals which included alkaloids, carbohydrates, glycosides, phytosterols, proteins, flavonoids, tannins, saponins, phenols and terpenes by employing the standard methods (El-Nour, 2002; Evans, 1989).

**Antioxidant activity**

The antioxidant efficacy of methanol extract of seeds of MC was evaluated by DPPH-free radical scavenging activity by means of the approach of Brand-Williams et al., (Brand-Williams et al., 1995), where ascorbic acid was employed as standard**.**

**Cytotoxicity activity**

This experiment was conducted on brine shrimp nauplii. Four distinct test solutions were used to evaluate the cytotoxicity of the extract, where vincristine sulfate used as a positive control (McLaughlin et al., 1998).

**Thrombolytic activity**

Thrombolytic activity was conducted on the basis of method described by Furie and Furie (Furie and Furie, 2008), where streptokinase solution was used as standard.

Difference obtained in weight taken before and after clot lysis was expressed as percentage of clot lysis as shown below: 

% of clot lysis= (wt. of released clot /clot wt.) ×100.

**Antibacterial screening**

The crude extracts (from which natural compounds were isolated) were tested for possible antimicrobial activity by using the disc diffusion technique (Chowdhury et al., 2015).

**Analgesic activity test**

Acetic acid-induced writhing method was used to check the analgesic effect of the extract (Ahmed et al., 2001). In the present study, diclofenac sodium was used as a standard drug. 

**Anthelminthic test**

The anthelmintic assays were executed as per the approach of Ghosh et al. with minor modifications (Ghosh et al., 2005). Albendazole was used as reference standard.

**Antidiarrheal activity test**

Castor oil-induced diarrhea was induced according to method of Uddin et al. (Uddin et al., 2005).

**Open field test**

This test was used to find out exploratory activity under identical situations. The test was conducted according to the method of Gupta et al., (Gupta et al., 1971). 

**Elevated plus-maze test**

This test has been widely used to understand and determine the anxiolytic- and anxiogenic-like activities in rodents (Woode et al., 2011; Sen et al., 2018). The entire test was carried out in a sound attenuated room. 

**Statistical analysis**

SPSS software package, version 16.0 (SPSS, Inc. Chicago, IL) was used to analyze the data. Values are expressed as mean±SEM. Comparison of all parameters of all the subjects was made by one-way ANOVA using SPSS software. The significance level was considered at p < 0.05.

## Results

**Phytochemical screening**

The phytochemical screening of crude methanolic extract showed positive response for the presence of alkaloids, carbohydrates, glycosides, saponins, phytosterols, phenols, tannins, flavonoids, proteins and amino acids. It produced a negative (-) response for terpenes in copper acetate test. The result of preliminary phytochemical screening is represented in Table 1.

**Antioxidant activity**

The half maximum inhibitory concentration (IC50) was 114.39 μg/ml and the IC50 value for standard ascorbic acid was 0.41 μg/ml.

**Cytotoxicity activity**

In this study, the positive control (vincristine sulfate) exhibited LC50 value at a concentration of 0.1812 µg/ml where methanolic extract, chloroform fraction, n-hexane fraction had LC50 of 26.40 µg/ml, 37.08 µg/ml and 17.85 µg/ml, respectively (Table 2).

**Table 1:** Phytochemical screening of seeds of MC

Sl. No.	Phytochemicals	Test	Methanolic extract
	Alkaloids	Wagner’s test	**+**
		Hager’s test	**+**
	Carbohydrates	Fehling’s test	**+**
		Benedict’s test	**+**
	Glycosides	Legal’s test	**+**
	Saponins	Froth test	**+**
	Phytosterols	Salkowski’s test	**+**
		Libermann-Burchard’s test	**+**
	Phenols	Ferric Chloride test	**+**
	Tannins	Ferric Chloride test	**+**
	Flavonoids	Alkaline reagent test	**+**
	Proteins and amino acids	Xanthoproteic test	**+**
	Terpenes	Copper acetate test	**+**

(+) = Presence of phytochemicals and (-) = Absence of phytochemicals

**Table 2:** Cytotoxic activity of different fractions of seed of MC

Sample	LC_50_ (µg/ml)	Regression equation	R^2^
**Vincristine sulfate (Positive control)**	0.1812	y=20.022x+64.857	0.8902
**Methanolic extract**	26.40	y = 34.471x+0.9898	0.941
**Chloroform fraction**	37.08	y=32.594x-1.1433	0.9432
**Carbon tetrachloride fraction**	83.42	y=28.84x-5.4096	0.8703
**n-hexane fraction**	17.85	19.795x+25.222	0.8832

**Thrombolytic activity**

We found that blood clot lysis activity of methanolic extract of MC was concentration-dependent, lowest at the concentration of 2 mg/ml (14.88 %) and remarkable at the concentration of 10 mg/ml (46.12%) when compared to the positive control streptokinase (Table 3).

**Antibacterial activity**

Methanolic extract of MC was investigated by determining the zone of inhibition. In this study, *Bacillus subtilis* and *Staphylococcus aureus* were taken as Gram positive (+) while *Escherichia coli* was taken as Gram negative (-) and tetracycline was taken as standard. 

In this screening work, the crude methanolic extract produced considerable zone of inhibition for Gram positive (+) *S. aureus* (15 mm in 1000 µg/ml) and Gram negative (-) *E. coli* (13 mm in 1000 µg/ml) when compared to the standard tetracycline (30 mm and 22 mm, respectively). Surprisingly, it did not produce any kind of antimicrobial properties against Gram positive (+) *B. subtilis *(Table 4).

**Table-3:** Thrombolytic activity of methanolic extract of seeds of MC

Sample Name	Concentration. (mg/ml)	Clot Lysis Mean ± SEM
ME1	10 mg/ml	46.12±1.59
ME2	8 mg/ml	40.64±2.35
ME3	6 mg/ml	34.79±1.75
ME4	4 mg/ml	31.89±1.27
ME5	2 mg/ml	14.88±1.19

ME stands for methanolic extract, each value represents the mean±SEM, n=5.

**Table 4:** Antibacterial activity of methanol extract of seed of MC

Test organisms	Diameter of zone of inhibition (mm)					
	Methanol extract					Standard
	1000 µg/ml	100 µg/ml	10 µg/ml	1 µg/ml		Tetracycline (30 µg/disc)
Gram positive bacteria						
*B. subtilis*	-	-	-	-	23	
*S. aureus*	15	12	10	7	30	
Gram negative bacteria						
*E. coli *	13	11	6	5	22	

**Analgesic activity**

The different fractions of seed extract of MC exhibited dose-dependent inhibition of acetic acid-induced writhing in mice in comparison to the standard. In the study, it was found that methanolic and chloroform extract at a dose of 400 mg/kg body weight showed promising analgesic activity when compared to the standard (78.35%) (Table 5).

**Anthelminthic activity**

Additionally, we found a dose-dependent anthelmintic property for this plant extract in the *in vitro* anthelmintic assay against adult earthworm (Table 6). The paralysis time at different concentrations of 10, 20, 30, 40 and 50 mg/ml was 52.6, 43.4, 34.4, 22.2, 16.6 min, respectively whereas death time at the same concentrations was 77.4, 62, 48.2, 31.4, 27.6 min, respectively. 

**Neurological activity test**

In open field test, at 400 mg/kg, methanol crude extract, chloroform fraction, carbon tetra-chloride fraction and n-hexane fraction showed no exploratory effect in mice at 90, 60, 30 60, 90 min respectively, while standard diazepam showed significant activity at all doses level (Table 8).

**Table 5:** Analgesic activity of methanolic crude extract and its different fractions of MC seeds

Group	Number of writhing (Mean±SEM)	% of inhibition of writhing
**Control**	25±0.45	_
**Standard (Diclofenac sodium)**	8.4±0.75	78.35
**Methanol (crude) extract** **(400 mg/kg body weight)**	15.8±0.38***	36.80
**Chloroform fraction** **(400 mg/kg body weight)**	20.8±0.86***	16.80
**Carbon tetrachloride fraction** **(400 mg/kg body weight)**	23±0.84	8.69
**n-hexane fraction** **(400 mg/kg body weight)**	22.8±0.38	8.80

Each value represents the mean±SEM, n=5. ***p<0.001 compared with the control.

**Table 6:** Time for paralysis and death of earthworms for extract and standard

Group	Concentration (mg/ml)	Paralysis time (min) Mean±SEM	Death time (min) Mean±SEM
Sample 1	10	52.6 ± 0.501	77.4 ± 0.748
Sample 2	20	43.4 ± 0.678	62 ± 0.837
Sample 3	30	31.4 ± 0.60	48.2 ± 0.374
Sample 4	40	22.2 ± 37.2	31.4 ± 0.501
Sample 5	50	16.6 ± 0.576	27.6 ± 0.812
Standard	15	43.6 ± 0.678	62 ± 0.548
Control	0	_	_

Values are expressed as mean±SEM, N=5

**Antidiarrheal activity test**

In castor oil-induced diarrhea test, n-hexane fraction showed promising anti-diarrheal effect in mice with diarrheal inhibition of 44.58%. Methanol extract at dose of 200 and 400 mg/kg body weight moderately inhibited the frequency of defecation (30.80% and 44.42%) when compared with untreated control mice (p<0.05). (Table 7).

In elevated plus maze test, each extract- methanol crude extract, chloroform fraction, carbon tetra-chloride fraction, n-hexane fraction at 400 mg/kg showed no neurological activity at both conditions (% of number entry into open arm and % of time spent in open arm) (p<0.05) (Table 9).

**Table 7:** Effect of different fractions of crude extract of seeds of MC on castor oil-induced diarrhea

Groups	Treatment (p.o.)	Total number of feces	% Inhibition of defecation	Total number of diarrheal feces	% Inhibition of diarrhea
I	Saline (2 ml/kg)	21±1.00	-	12±0.57	-
II	Loperamide (5 mg/kg)	8.76±0.56***	51.82	5.55±0.43***	44.78
III	Methanolic extract (200 mg/kg)	13.67±0.88**	34.90	8.33±0.88**	30.58
IV	Methanolic extract (400 mg/kg)	12.68±1.20**	36.61	6.67±0.88**	44.42
VI	Chloroform fraction (400 mg/kg)	16.67±1.20*	20.62	8.67±0.88*	27.25
VII	Carbon tetrachloridefraction (400 mg/kg)	17±0.57*	19.04	9±0.573*	25
VII	n-hexane fraction (400 mg/kg)	9.33±0.881**	58.71	5.65±0.881**	44.58

Values are expressed as mean±SEM (n=5). *p<0.05 **p<0.01, and ***p<0.001 show significant differences when compared with the control group.

**Table 8:** Screening of open field test activity of different fractions and crude extract of seeds of MC

Groups	Dose (p. o.) (mg/kg)	No. of movements			
		**0 min**	**30 min**	**60 min**	**90 min**
Control	0.5 ml/mice	278.3±.33	284±0.578	290.04±1.0	298.33±1.45
Diazepam	1	130.5±5.62	125.7±1.23***	119.68±3.45***	115.56±4.21***
Methanolic extract	400	275±0.06	283±1.15	287.00±0.57	296±3.05*
Chloroform fraction	400	277.7±1.45	280.3±0.88**	289±1.52	295.87±0.66
CCl_4_ fraction	400	275±2.64	282.7±1.85	286±0.57*	293.67±1.45
n-hexane fraction	400	278.7±0.66	283±0.33	289±0.57	296.33±1.85**

Values are expressed as mean ±SEM, N=5. *p<0.05, **p<0.01, and ***p<0.005 show significant differences compared with the control.

**Table 9:** Effect of MC seeds methanol extract and its different fractions in EPM test during 5 min test session

Groups	Dose (p. o.) (mg/kg)	% of number entry into open arm	% of time (in seconds) spent in open arm
Control	0.5 ml/mice	53±1.05	49.6±0.509
Diazepam	1	70.8±0.67***	77.6±1.03**
Methanolic extract (crude)	400	52±0.71**	47.4±0.81*
Chloroform fraction	400	51±0.84**	46.8±0.66*
CCl_4_ fraction	400	51±0.71*	49.2±0.56*
n-hexane fraction	400	50.4±0.51**	47.8±0.75*

Values are represents in mean±SEM, N=5. *p<0.05, **p<0.01, and ***p<0.005 show significant differences compared with the control.

## Discussion

Medicinal plant materials have become an interesting source for both conventional and contemporary medicines, and herbal medicines has been shown to have genuine utility in the field of medicine (WHO, 1978; Hussain et al., 2018). The aim of our present study was to investigate general *in vitro* and *in vivo* bioactivities of seeds of MC. In preliminary phytochemical screening, crude methanolic extract of MC showed positive (+) response for the presence of alkaloids, carbohydrates, glycosides, saponins, phytosterols, phenols, tannins, flavonoids, proteins and amino acids. In the course of antioxidant assay, the DPPH radical activity of the methanolic extract was found to be increased with the increasing concentration. Agents which are used for the management of cancer show toxicity on normal cells in particular rapidly growing cells (Terrence, 2008). To investigate the cytotoxic activity, brine shrimp lethality assay is one of the most convenient, accessible and inexpensive bioassay and this method correlates in most cases reasonably well with cytotoxic and antitumor properties (Sonibare et al., 1995). From the result of the brine shrimp lethality bioassay, it was observed that the % of the morbidity is proportional to the concentration i.e. % of the morbidity was increased with the increasing concentration of the extract. 

Thrombolytic agents commonly lyse clot via disrupting the fibrin and fibrinogen (Das et al., 2013; Abedin et al., 2018; Hussain et al., 2016). In our study we assessed whether the crude methanolic extract possesses any clot lysis potential or not. We found that thrombolytic activity of methanolic extract of MC was concentration-dependent, lowest at the concentration of 2 mg/ml (14.88%) and remarkable at the concentration of 10 mg/ml (46.12%). Phytochemicals present in the medicinal plants, like saponins, alkaloids and tannins are responsible for the thrombolytic activity (Das and Dewan, 2013) and as methanolic extract of MC contain considerable amounts of these phytochemicals they can be the probable motive for the thrombolytic activity. 

 Nowadays, the alarming occurrence of antibiotic resistance in bacteria is a medical significance and necessitates brand new and effective therapeutic agents that can strongly fight microbial agents (Agrawal et al., 1996; Parekh et al., 2005). In our present work, antimicrobial properties of methanolic extract of MC were investigated by determining the zone of inhibition. The concentration of 1000 μg/ml was found to resistant on Gram positive (+ve) *Bacillus subtilis* but produced significant antimicrobial activity against Gram positive (+ve) *Staphylococcus aureus* and Gram negative *E. coli* (zone of inhibition was 15 and 13 mm, respectively). 

It was observed from the previous studies in analgesic activity assay model the plant extract demonstrated analgesic effects. The examined fraction of MC remarkably and dose-dependently inhibited acetic acid-induced writhing in mice when compared to the standard. Acetic acid generally induces inflammatory pain via arousing capillary permeability (Amico-Roxas et al., 1984), and liberating substances that are responsible for the excitement of pain nerve endings (Raj, 1996). The peripheral analgesic effect is commonly mediated through NSAIDs by the inhibition of COX and/or LOX (and different inflammatory mediators) or inhibition of ache responses mediated by nociceptors peripherally (Koster et al., 1959). Therefore it is far feasible that crude methanolic extract can display analgesic potentiality through those mechanisms although the precise mechanism of motion is needed to be determined. 

Also, it was determined that crude methanolic extract exhibited potentiality of anti-diarrheal action. Loperamide is broadly used for the control of diarrhoeal diseases which efficaciously antagonizes diarrhea precipitated by the castor oil (Al-Taher, 2008). In case of antidiarrheal activity test, aqueous fraction (400 mg/kg body weight) showed considerable percentage of diarrheal inhibition. It was reported that the presence of tannins, alkaloids, saponins, flavonoids and triterpenes are responsible for anti-dysenteric and anti-diarrhoeal properties of a plant extract (Kolawole et al., 2010). This can be because of the fact that the extract can increase the reabsorption of water by lowering intestinal motility within the isolated rabbit ileum. Phytochemical screening found out the presence of flavonoids, tannins, saponins, cardiac glycosides, alkaloids, carbohydrates, proteins and amino acids. Hence, tannins are well known for their lowering impact on GI motility among the phyto-constituents (Galvez et al., 1993).

Phytochemical analysis of the methanolic extract confirmed the presence of phenols, tannins, saponins that show anthelmintic property. According to a previous study, by uncoupling oxidative phosphorylation, the phenolic compounds can interfere with the helminthic parasites (Athanasiadou et al., 2001) and phenolic compounds additionally bind to the loose proteins in the gastrointestinal tract of host animal and glycoprotein on the cuticle of the parasite which ultimately lead to the parasite on dying (Tiwari et al., 2011). 

In the central nervous system, gamma-aminobutyric acid (GABA) is the major inhibitory neurotransmitter (Ripa et al., 2015) and it is evidence that many anxiolytic, muscle relaxants and sedative-hypnotic drugs exert their actions via GABA (Angad et al., 2010). Some previous researchers in this area showed that phytoconstituents like flavonoids, act as ligands for the GABAA receptors in the central nervous system, which led us to a major hypothesis that these flavonoids may act as benzodiazepine-like molecule (Ripa et al., 2015). Therefore, it may be suggested that the plant extract may exert its neuro-pharmacolo

gical action by potentiating GABAergic inhibition in the CNS via membrane hyperpolarization (Angad et al., 2010; Kolawole et al., 2010). The phytochemical assessment showed the presence of alkaloids, flavonoids, saponins and steroids in the plant (Kavitha et al., 2011; Kamal, 2014) but it was a surprising fact that the crude methanolic extract did not exhibit any neuro-pharmacological activity in EPM and open field test. This requires a further investigation to figure out the cause.

Based on the present study, it can be concluded that the seed extract of MC possesses antioxidant, cytotoxic, thrombolytic, antimicrobial, analgesic, antidiarrhoeal, anthelmintic effects which led us to the inference that the plant extract may contain bioactive compounds. Qualitative tests revealed the fact that methanolic extract of seed of MC contains major phytochemicals viz. carbohydrate, glycosides, phenolics, flavonoids, phytosterols, tannins, proteins and amino acid, saponins, terpenoid and alkaloids. Our present study was entirely focused on the seed value. It is thought worthy to select this extract for further studies.
